# Non-contact ultrasonic inspection by Gas-Coupled Laser Acoustic Detection (GCLAD)

**DOI:** 10.1038/s41598-021-04191-x

**Published:** 2022-01-07

**Authors:** Michelangelo-Santo Gulino, Mara Bruzzi, James Norbert Caron, Dario Vangi

**Affiliations:** 1 Department of Industrial Engineering of Florence, Università degli Studi di Firenze, Via di Santa Marta 3, 50139 Florence, Italy; 2 Department of Physics and Astronomy, Università degli Studi di Firenze, Via Sansone 1, 50019 Sesto Fiorentino, Italy; 3 Research Support Instruments, 4325-B Forbes Boulevard, Lanham, MD 20706 USA

**Keywords:** Mechanical engineering, Optical sensors

## Abstract

Gas-Coupled Laser Acoustic Detection (GCLAD) is an ultrasonic, non-contact detection technique that has been recently proven to be applicable to the inspection of mechanical components. GCLAD response raises as the intersection length between the probe laser beam and the acoustic wavefront propagating in the air increases; such feature differentiates the GCLAD device from other optical detection instruments, making it a line detection system rather than a point detector. During the inspection of structures mainly extending in two dimensions, the capability to evidence presence of defects in whichever point over a line would enable moving the emitter and the detector along a single direction: this translates in the possibility to decrease the overall required time for interrogation of components compared to point detectors, as well as generating simpler automated monitoring layouts. Based on this assumption, the present study highlights the possibility of employing the GCLAD device as a line inspection tool. To this end, preliminary concepts are provided allowing maximization of the GCLAD response for the non-destructive testing of components which predominantly extend in two dimensions. Afterwards, the GCLAD device is employed in pulse-echo mode for the detection of artificial defects machined on a 12 mm-thick steel plate: the GCLAD probe laser beam is inclined to be perpendicular to the propagation direction of the airborne ultrasound, generated by surface acoustic waves (SAWs) in the solid which are first reflected by the defect flanks and subsequently refracted in the air. Numerical results are provided highlighting the SAW reflection patterns, originated by 3 mm deep surface and subsurface defects, that the GCLAD should interpret. The subsequent experimental campaign highlights that the GCLAD device can identify echoes associated with surface and subsurface defects, located in eight different positions on the plate. B-scan of the component ultimately demonstrates the GCLAD performance in accomplishing the inspection task.

## Introduction

The application of non-contact ultrasonic excitation and detection techniques are now standard practice in the non-destructive inspection of mechanical components, structures and systems. The most typical of these techniques are based on excitation via pulsed lasers and optical detection via vibrometers or interferometers, as well as excitation/reception by electromagnetic acoustic transducers (EMATs) in conductive materials^1^ or piezoelectric Air-Coupled Transducers (ACT). The ability to excite and detect ultrasonic waves without coupling means makes these techniques advantageous over more traditional contact methods^2^. In fact, they avoid contamination of the component’s surface^3^, to operate in aggressive environments (corrosive or at elevated temperatures^4,5^), to prescind from the piece geometry and from any limitation in the accessibility and finish of the surfaces^6^, to monitor moving parts during their operation^7–9^. Moreover, these techniques often allow speeding up the inspection process by adopting flexible movement patterns for the detector^10^, a particularly desirable condition in sectors such as aeronautics where the control of extensive aircraft structures has become a maintenance practice. In this landscape, optical techniques appear to be conceivably the most promising ones because of their versatility, good sensitivity and resolution, the wide frequency band of the signal they can generate and receive, and their applicability to almost all types of material. The excitation of ultrasound is quite easy to obtain with pulsed lasers^11–14^ or continuous modulated lasers^15,16^, using the photoacoustic effect in elastic or ablation regime (plasma-generated ultrasound), applicable practically both on any material of engineering interest and on various types of surfaces, being rough, smooth, reflective or opaque.

The detection of ultrasonic waves with optical techniques features a high level of complexity: many technologies are sensitive to the degree of finish/reflectivity of the surface and currently require expensive instruments, not always suitable for use in industrial environments. In recent years, however, many steps forward have been made and tools are presently available that can detect ultrasonic waves even in the presence of non-reflective surfaces and with a certain roughness. Interferometers (Michelson’s variant, in their simplest form^17,18^) are based on the interference between the optical beam emitted by the instrument and the optical beam reflected from the surface to be inspected. Vibrometers represent a particular type of interferometers based on the Doppler effect (laser Doppler vibrometers^19^): for effective ultrasound detection, the emitted laser beam is immediately split into two beams, one with an additional frequency component resulting from the passage through an acousto-optic modulator; the differences between the two beams in frequency terms, more evident because of the preliminary passage in the modulator, provide the speed of the particles on the surface rather than their displacement. Interferometric techniques are characterized by high sensitivity, broad band and point detection^20^. Optimization of the tools made these techniques usable even on not perfectly smooth or reflective surfaces^21,22^, finding applications in numerous industrial fields. Alongside these techniques and tools, there are less established technologies like the Optical Beam Deflection (OBD)^23^, where a laser probe beam is incident on the surface of the piece with a certain angle. The laser beam is reflected from the surface at an angle that depends on the shape of the surface itself, altered by the passage of the ultrasonic wave. Using a spatial filter (knife-edge), part of the beam is intercepted, and the overcoming part determines changes in the light intensity that reaches a detection photodetector^24^. OBD is a point detection technique, and its response strongly depends on the quality of the piece surface reflecting the laser beam, effectively limiting its use in cases of industrial interest.

Another optical technique is the Gas-Coupled Laser Acoustic Detection (GCLAD), whose physical principle leverages on the deviation of a probe laser beam propagating in air or water, in correspondence of areas with variations in the refractive index^25^. When the ultrasonic waves propagating inside the component reach the surface, they are transmitted to the air, generating pressure waves that alter the refractive index. This causes the deviation of the probe beam, acquired by a position-sensitive photodetector. The sensitivity of the technique depends on how much the ultrasound is attenuated because of the transmission from the solid component to the air, as in a typical ACT. However, the displacement of the beam in correspondence of the photodetector is provided by the integral along the path of all the displacements sustained by the laser beam immersed in the variable pressure field. Consequently, in certain inspection configurations, the response can be high and even exceed that for a confocal Fabry–Pérot interferometer^26^. The frequency response of the technique can reach up to 20 MHz in fluids like water, but it commonly covers ranges up to 8 MHz in air^27^; furthermore, since the laser beam does not interact directly with the surface of the piece, the response is independent of the optical properties and not particularly sensitive to the surface finish of the component^28^.

The GCLAD technique has currently few industrial applications and there is no evidence of implementation in NDT processes. The knowledge on the physical principles that regulate the detection, useful for optimizing the GCLAD technique for NDT applications, has recently been deepened in some studies by the authors^29,30^: the GCLAD system can be exploited in various experimental configurations, obtaining the maximum sensitivity in configurations where the interaction zone between the probe laser beam and the ultrasonic wave refracted in air is maximized. The study also illustrates how the GCLAD system allows detecting Surface Acoustic Waves (SAWs) refracted in the air from any point belonging to a line on the surface. The presence of a defect anywhere on this line generates a signal that can be detected by the system. This makes the GCLAD a line inspection tool rather than a point detection device, with consequent advantages in terms of defect detection capabilities. A defect with depth, width, and thickness significantly lesser than the ultrasonic wavelength is overcome by the oscillations: following the acquisition by a point detection device, ultrasonic waves will exhibit the same signal amplitude independently of the defect presence, should the defect be positioned far from the detection location^31^. The defect can hence be identified only if the transducer is moved along an entire line starting from the ultrasonic emission location, until the transducer position corresponds to that of the defect. With a line inspection tool as the GCLAD, such line can be entirely scanned at once without moving the apparatus, since whichever discontinuity in the material surface translates in a discontinuity in the acquired ultrasonic signal^32^. Currently, these features of the GCLAD have been preliminarily investigated on specimens, but never applied to perform line inspections on mechanical components.

The objective of the present work is to pinpoint the characteristics of the GCLAD technique in line inspections, useful for speeding up the ultrasonic monitoring processes of wide surfaces. For this purpose, application for SAW detection and identification of surface defects on an element chiefly extending in one dimension (i.e., a bar) is first illustrated, both in pitch-catch and pulse-echo mode. Subsequently, an application to the control of a two-dimensional plate with surface defects is reported, where the scanning of the whole component takes place with the system moving along a single direction. Finally, the effectiveness of the technique is also highlighted in detecting subsurface defects^33^, introduced in the same plate.

## GCLAD technique

The principle behind the probe beam deflection or Gas-Coupled Laser Acoustic Detection (GCLAD) technique^26,34,35^ is the deflection and displacement of a probe laser beam when it crosses a fluid region which is subjected to refractive index fluctuations. Such fluctuations can be associated with a variable pressure field triggered by the propagation of ultrasonic waves inside the fluid domain^36^. Total deviation of the probe laser beam is acquired by a photodetector and transduced into a proportional voltage, which has been thoroughly investigated by Caron^26^.

To explain the GCLAD functioning, a pressure field with sinusoidal profile and generated by an acoustic wave which propagates along the *z* axis is preliminarily referred to:

1 $$\begin{aligned} p(z, t)=k \rho _{0} \nu ^{2} \delta \sin (k z-\omega t). \end{aligned}$$

**Figure 1:**
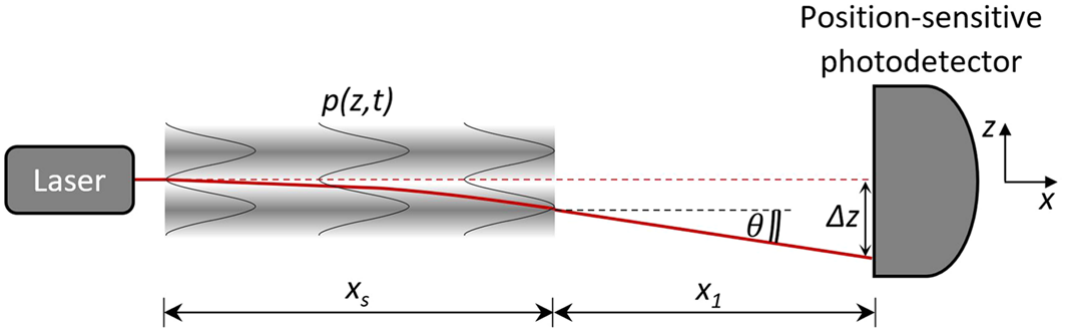
The ultrasonic pressure field *p*(*z*, *t*) generates a refractive index variation in an $x_s$-long fluid region; the GCLAD laser beam sustains deflection and displacement in such region, propagating unperturbed afterward along a $x_1$-long path. The position-sensitive photodetector identifies the overall $\varDelta z$ displacement and transduces it into a voltage.

In Eq. (1), $\omega$ is the acoustic wave angular frequency in the fluid, $\rho _0$ is the density of the unperturbed fluid, $\nu$ the wave speed in the fuid, *k* the wavenumber $\omega /\nu$, *z* its distance from the surface of the component and $\delta$ the displacement of the surface itself (that generates the ultrasound in the fluid). Referring to Fig. 1, based on the eikonal equation^37^, the displacement $\varDelta z$ of the optical ray which travels along the *x* axis in an ortogonal direction with respect to a pressure wave *p*(*z*, *t*) can be obtained in correspondence of the photodiode:

2 $$\begin{aligned} \varDelta z=\frac{2 \pi ^{2}\left( n_{0}-1\right) }{\nu ^{2} n_{0}} \delta f^{2} x_{s}^{2} \cos \left( k z_{0}-k \nu t\right) +\theta x_{1}. \end{aligned}$$

In Eq. (2), $n_0$ is the unperturbed fluid refractive index, *f* its frequency, $x_s$ the length of the acoustic pressure field and $\theta$=*d*($\varDelta z$)/$dx_s$ the deflection angle.

The deviation of the probe beam is acquired by a position-sensitive photodetector constituted of two photocells and transduced in a proportional voltage difference^26^:

3 $$\begin{aligned} \varDelta V=\frac{-8 \mathrm {GR} \kappa P}{\sqrt{\pi ^{3}}} erf\left( \frac{d}{w}\right) \frac{\varDelta z}{w}\left[ e^{\left( -\frac{d^{2}}{4 w^{2}}\right) }-e^{\left( -\frac{t^{2}}{4 w^{2}}\right) }\right] , \end{aligned}$$

where *G* is the amplifier gain, *R* is the resistance employed to convert the current from the photodiode to voltage difference, *P* the average power of the laser beam at the photodetector, *w* is the diameter of the probe laser beam, *d* is the diameter of the active area of the photodetector, *t* the separation distance between the cells, and $\kappa$ is the photodetector sensitivity. Based on Eqs. (2) and (3), response increases as the length of the acoustic pressure field $x_s$ and the distance from the photodiode $x_1$ are increased.

**Figure 2:**
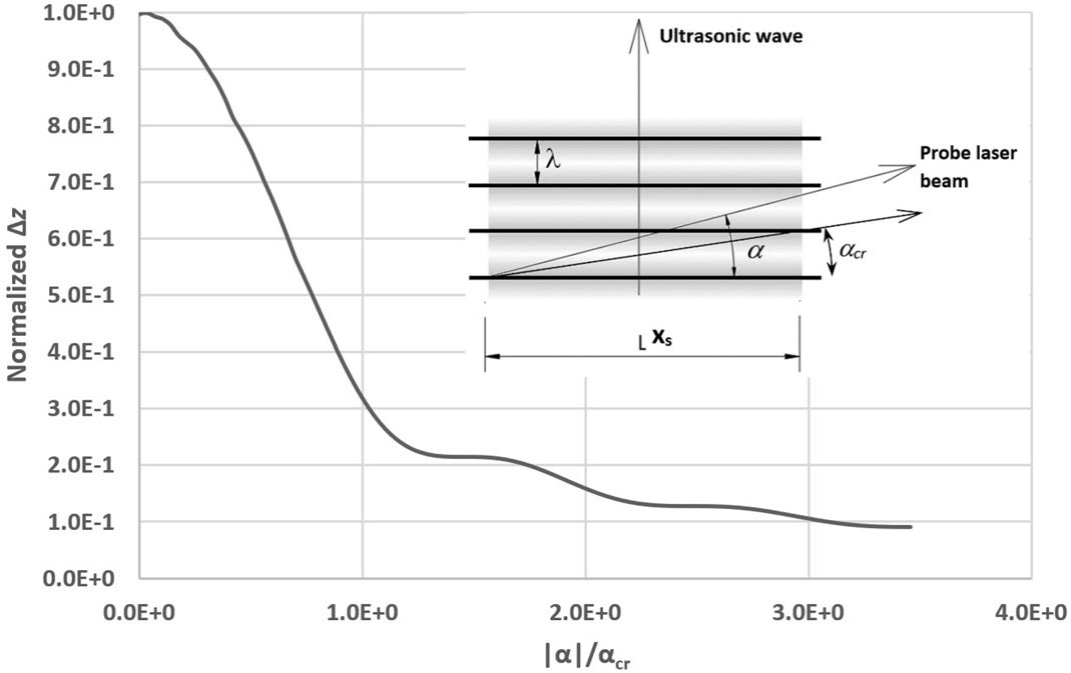
Amplitude of the probe laser beam total deviation along *z* ($\varDelta z$) normalized to its maximum, based on the value of the propagation angle $\alpha$ normalized to the critical angle $\alpha _{cr}$ (modified from Gulino et al.^29^).

Equation (2) refers to parallel directions for probe laser beam and wavefront. Assuming that a finite angle $\alpha$ exists between such directions (Fig. 2), the GCLAD response consequently modifies: the beam encounters a variable pressure gradient, so that $\varDelta z$ equals the integral of all deviations the laser beam sustains while travelling along $x_s$. Let us indicate with $\alpha _{cr}$ the angle represented by $\alpha _{cr}$ = tan^−1^($\lambda /x_s$), where $\lambda$ is the ultrasonic wavelength. $\alpha _{cr}$ is generally lesser than one degree; Fig. 2 shows the theoretical value of $\varDelta z$ as the ratio of $\mid$ $\alpha$ $\mid$/$\alpha _{cr}$ is modified (the trend is the same for positive or negative values of $\alpha$^29^): the amplitude of $\varDelta z$ rapidly decreases when a significant difference exists between laser beam orientation and propagation direction of the acoustic wavefront. The system is hence selective to the propagation direction of the wavefront, being able to mainly detect pressure waves in perpendicular directions.

**Figure 3:**
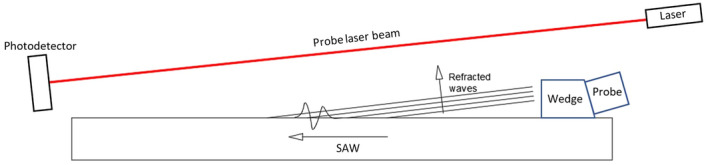
Experimental setup which enable maximizing the GCLAD response for ultrasound detection.

Therefore, to obtain the maximum sensitivity in the visualization of the ultrasonic waves, it is convenient to employ an experimental configuration in which the laser beam is parallel to the wavefront refracted in the air. The layout hence enables identification of patterns associated with specific acoustic waves, namely the direct (pitch-catch) or the reflected ones (pulse-echo) also in a context of defect detection^30^. For example, Fig. 3 depicts the experimental arrangement for detecting a surface wave propagating on a plate with maximum sensitivity. The plate is made of S355 steel, has a thickness of 12 mm, a length of 520 mm, and a width of 70 mm. The laser beam is inclined relative to the solid surface for making it normal to the propagation direction of the refracted Rayleigh wave. An identical configuration can be used to detect bulk waves, employing a diverse angle between the probe laser beam and the surface because the refracted waves feature a diverse propagation direction. To maximize response, the beam must pass as close as possible to the surface to be interrogated (amplitude halves when the distance from the surface doubles^32^). The minimum vertical distance between the specimen surface and the beam has been set as 110 mm to avoid conflict among positions of the laser source, the photodetector, and the ultrasonic probe. Such distance has been applied to all reported experiments.

**Figure 4:**
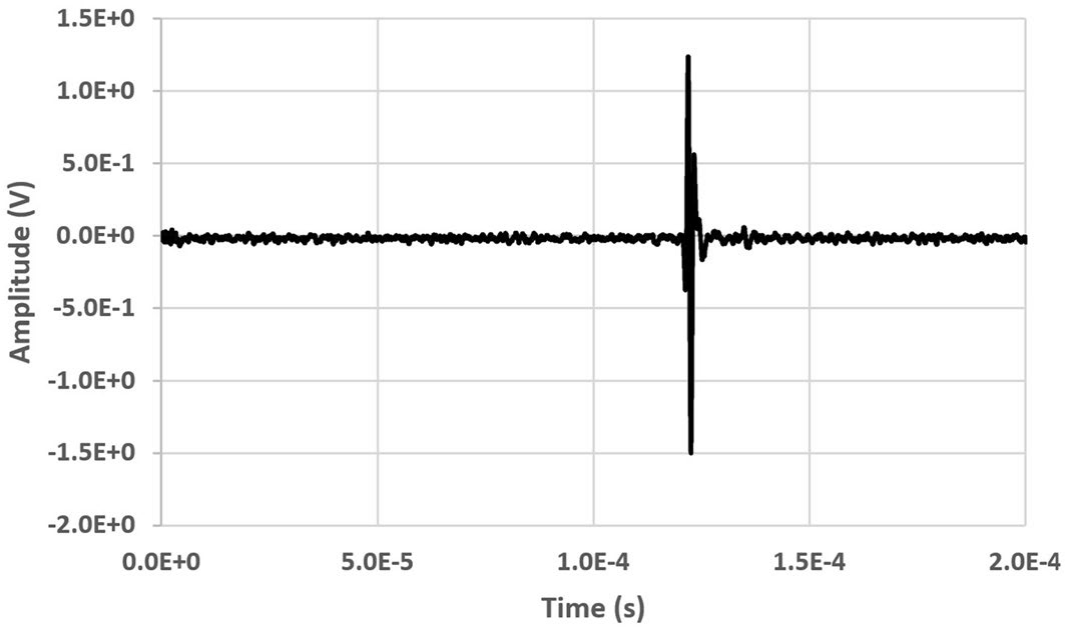
Signal from a surface wave propagating in the component as in the scheme of Fig. 3, refracted in air and subsequently detected by the GCLAD device.

As an example, Fig. 4 shows the signals received with GCLAD in the configuration of Fig. 3, in case of a SAW generated by a contact probe with central frequency of 1 MHz, $x_s$ = 280 mm, $x_1$ = 100 mm, and a diameter of the laser beam equal to 1 mm, obtaining a signal to noise ratio (*S*/*N*) of 35 (32 ensemble averages). Based on the mutual inclination between the probe and the wedge, among the different types of SAW, Rayleigh waves are induced in the specimen^38^; because of the high ratio between the bar thickness and the acoustic wavelength in the metal (higher than two), excitation by the probe does not produce Lamb waves^39,40^.

**Figure 5:**
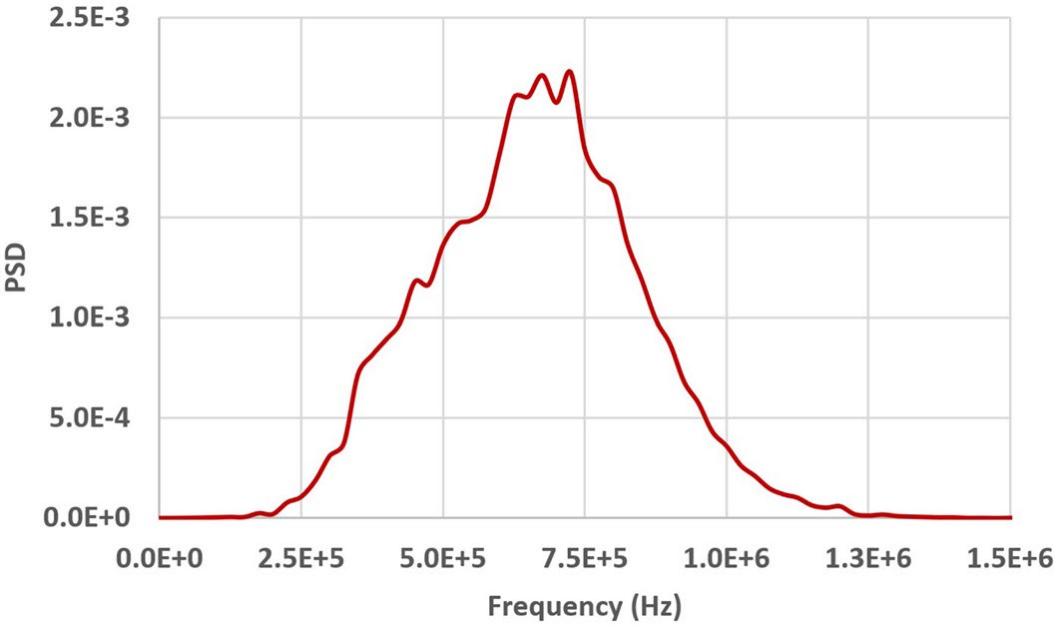
Power spectrum density (PSD) of the GCLAD signals in Fig. 4, obtained from a signal generated by a 1 MHz piezoelectric probe.

Figure 5 depicts the power spectrum density (PSD) of Fig. 4 signal acquired by the GCLAD. As can be seen, the GCLAD interprets the signal from the 1 MHz piezoelectric probe as a broadband ultrasound with a central frequency equal to 700 kHz. This effect depends on the finite spot of the probe laser beam compared to the acoustic wavelength. Equation (2) applies to infinitely small rays subjected to a variable pressure field at a quote *z*; if the beam has finite size, the infinitely small rays it is constituted of will occupate different *z* quotes; total deviation of the beam will be hence the sum of the deviations sustained by the constitutive rays, with a different overall form compared to the trend of Eq. (2); in particular, negligible interaction with acoustic wavelengths smaller than the beam spot will occur, resulting in no significant deviation for the probe laser beam (low pass filtering effect^29,41^). In the specific case, contents with higher frequencies will be relevantly attenuated because the laser spot (0.70 mm minor axis) approximately doubles $\lambda$ (0.34 mm).

**Figure 6:**
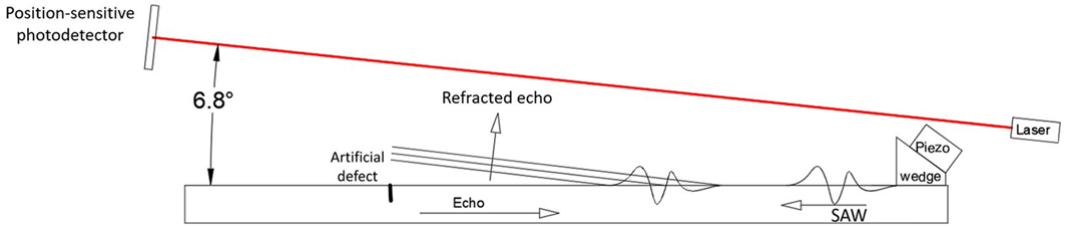
Scheme of experimental layout employed for the identification of defects in pulse-echo mode, with excitation of SAWs in the component by a contact piezoelectric probe and detection in air by the GCLAD device.

In case a defect is present reflecting the ultrasonic wave, the propagation direction of the ultrasound is mirrored both in the component and in the air. Figure 6 depicts a layout scheme for defect detection by SAWs in pulse-echo mode. The piezoelectric contact probe employed to induce SAWs in the rectangular-section bar has a central frequency of 500 kHz, while the probe beam is tilted of − 6.8$^\circ$ relative to the solid surface; this allows detecting solely the waves reflected by the backwall and the defect. The defect has a length of 15 mm and a depth of 3 mm (the depth almost equals the wavelength of the SAW in the bar). The distance of the probe beam from the specimen surface is 130 mm in correspondence of the defect.

**Figure 7:**
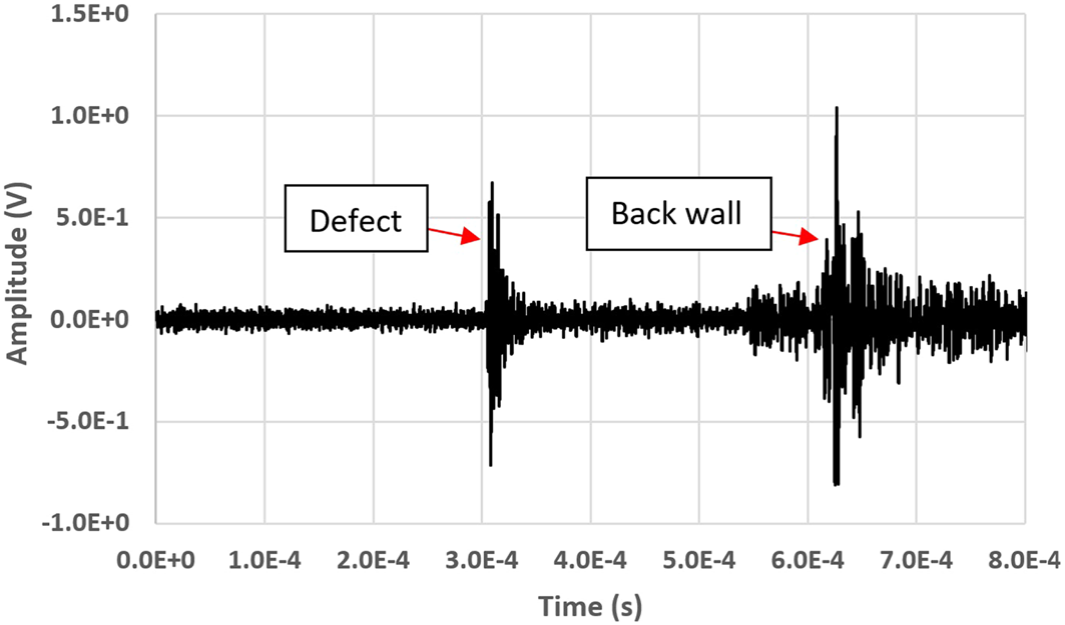
Echo signals from the defect and the back wall obtained in the configuration of Fig. 6.

Figure 7 depicts the echoes from the defect and the back wall, obtained employing a configuration in which $x_1$ = 540 mm and $x_s$ = 85 mm (32 ensemble averages). Also in this case, the distance between the specimen surface and the probe lase beam is minimized to increase the signal amplitude, consequently increasing the defect detectability. Since the defect depth is similar to the acoustic wavelength in the solid, part of the ultrasonic energy is unreflected by the defect and reaches the backwall. The signal associated with the backwall is consequently constituted of lower frequency components^42–44^. A similar signal can be obtained also by point detection devices as ACTs^30^ or vibrometers^22^, but their movement is required to scan the surface in search of defects whose dimensions are lower than the ultrasonic wavelength: the elastic waves are initially obstacled by the defect, but tend to regain their initial propagation pattern after a certain distance from the defect location^31^.

Figures 3, 4, 5, 6 and 7 evidence that the GCLAD device is efficient in highlighting ultrasonic waves which propagate on the specimen surface and are then refracted in air, as well as the presence of eventual surface defects. In this sense, the GCLAD does not differ from point detection devices in terms of defect identification: as a traditional piezoelectric probe or an ACT, the decrease in the signal amplitude and the time-of-flight can be used for defect sizing and localization respectively, in both pulse-echo or pitch-catch mode. It is however clear that the GCLAD acquired signals feature both a high amplitude and a low signal-to-noise ratio, the latter being the consequence of a relevant noise; the noise outputted by the detector is a combination of shot noise, dark current noise, thermal noise, laser intensity fluctuations and laser pointing instabilities. Additional noise from the circuitry or environment could affect the acquisition quality. Fine-tuning from all these diverse perspectives would be required to increase the acquisition quality. A previous research by the authors highlighted that, employing a 500 kHz central frequency piezoelectric probe as a source, a 500 kHz receiving ACT features an *S*/*N* that doubles the GCLAD one^30^. From a monitoring perspective and since the GCLAD device is extremely sensitive to vibrations, the best alternative to preserve the *S*/*N* is to move the component to be inspected, rather than the GCLAD device.

The detected signal amplitude depends on the considered value for the parameters *d*, $x_1$, $x_s$, but also on $\phi$. The liftoff distance *d* should be as low as possible, to mimize attenuation of the ultrasound during its propagation in air towards the probe beam. $x_1$ and $x_s$ must be conversely increased to obtain a proportional enhancement in the amplitude of the echo. For what regards $\phi$, it must be noted that a large beam spot makes the GCLAD device insensitive to high frequency components of the wave, but it contextually augments the photodetector response^26,41^.

## Materials and methods

The experimental layouts depicted in Figs. 3, 6 are functional to carry out an inspection on the line the probe laser beam insists on (i.e., line inspection). This can ease the automation of the monitoring setup, allowing to move the GCLAD and the source along a single direction to inspect a component which mainly extends in one or two dimensions. This would also result in a relevant decrease in inspection time compared to highly automated inspection methodologies as the Scanning Laser Source (SLS) or the Scanning Laser Line Source (SLLS) techniques: a pulsed laser source is moved along both directions in the defect proximity, evidencing the presence of a defect based on enhancements in the detected signal amplitude, determined by constructive interference between the direct surface waves and those reflected by the discontinuity^44^.

**Figure 8:**
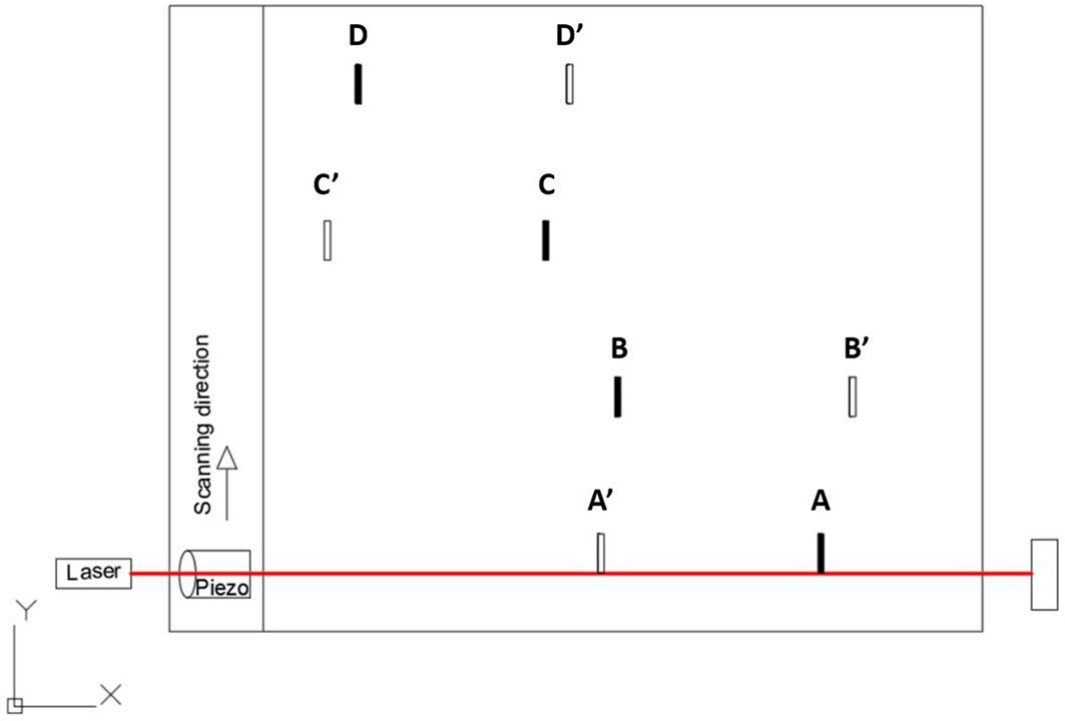
Plate with indications regarding position of the artificial defects: defects on the upper surface are depicted in black, in white on the inferior face.

As an application example of line inspection, the GCLAD technique has been applied to scan a metal plate on which artificial defects have been machined. The plate is in S355 structural steel, with a thickness of 12 mm and dimensions of 500 $\times$ 400 mm^2^. The defects have been triggered in the positions indicated in Fig. 8. The defects consist of an elliptical section notch with a thickness of 1 mm, with a depth (semi-minor axis) of 3 mm and a major axis of 20 mm. The defects have been obtained on both faces of the plate in different positions and at different times, to analyse the ability of the laser scanning technique to simultaneously detect defects on opposite faces.

**Figure 9:**
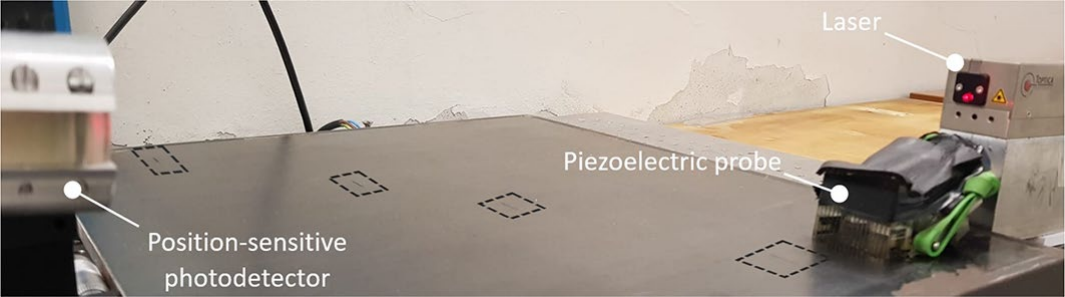
Experimental setup for the tests on the metal plate; presence of defect on the upper surface is highlighted by four black dashed rectangles.

In the scheme of Fig. 8, the projection of the GCLAD laser beam is oriented along the *x* axis; however, based on Fig. 6 or Fig. 8, the laser beam must also be perpendicular to the propagation direction of the SAW refracted by the metal in the air. The configuration used for the tests therefore requires the laser beam to be inclined of 6.8$^\circ$ relative to the plate surface, corresponding to the refraction angle of SAWs in the air for the considered plate. Because of the inclination, the laser beam in this case has a variable distance from the surface along its path. Exploiting the selectivity of the system, the inclination of the laser beam can be equal to + 6.8$^\circ$ or − 6.8$^\circ$ relative to the surface normal direction to detect respectively: (a) the wave propagating from the piezoelectric probe towards the piece or (b) the echo caused by a reflection on a defect. In this sense, like for air-coupled probes, it is possible to orient the beam to work in pitch-catch or pulse-echo mode. The inspection of the plate has been conducted in pulse-echo mode, i.e., with the laser beam oriented as shown in Fig. 6. The ultrasound propagating in the specimen is induced by a Panametrics contact piezoelectric probe with a central frequency of 500 kHz (600 kHz bandwidth) and a 25.4 mm diameter. For the excitation of SAWs, a Panametrics plexiglass wedge support is employed. A 2 mW TOPTICA iBeam Smart 640 laser is employed with a wavelength of 640 nm; the elliptical spot dimensions, equal to 1.2 $\times$ 0.7 mm^2^, are obtained by a beam profiler positioned at the exit of the laser source. The photodetector is a Red Enhanced Quad Cell Silicon Photodiode (SD 197-23-21-014, Luna Optoelectronics), and the acquired signals are pre-processed through a Brüel–Kjær 2638 wideband conditioner (filter from 300 kHz to 2 MHz, gain of 30 dB). The photodetector signal is acquired by an oscilloscope, triggered on the excitation of the piezoelectric probe so that the time scale origin corresponds to the emission of the acoustic wave at the probe/wedge interface. In Fig. 9, the experimental layout is highlighted.

## Results and discussion

**Figure 10:**
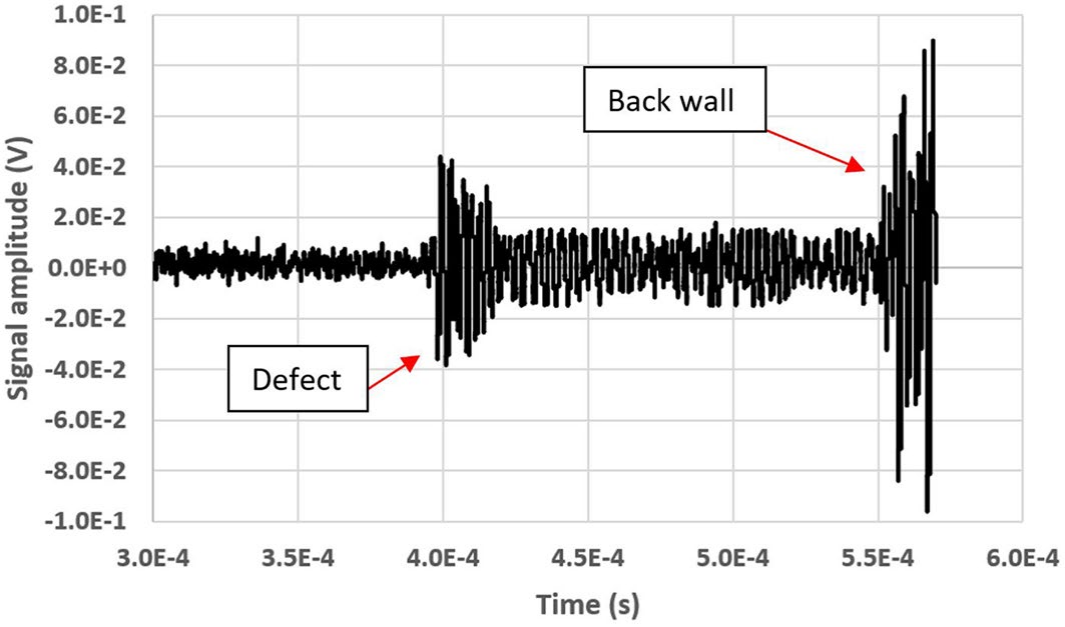
Signal acquired in correspondence of a surface defect. The signals associated with the defect and the the backwall echo are visible.

Initially, the scanning of the plate has been obtained by generating only defects on the upper surface. Figure 10 shows an example of signal acquired in correspondence of a defect (32 ensemble averages). The wave pattern caused by reflection on the defect and that due to the reflection on the backwall are clearly visible. The signal has a limited amplitude because of the high distance between the surface and the laser beam, which varies between 85 mm and 145 mm and resulting in higher attenuation to the ultrasonic wave. The low *S*/*N*, however, largely depends on the noise produced by the employed laser and on the electronics used to convert the signal into current from the bi-cell photodetector into voltage, which is not optimized.

**Figure 11:**
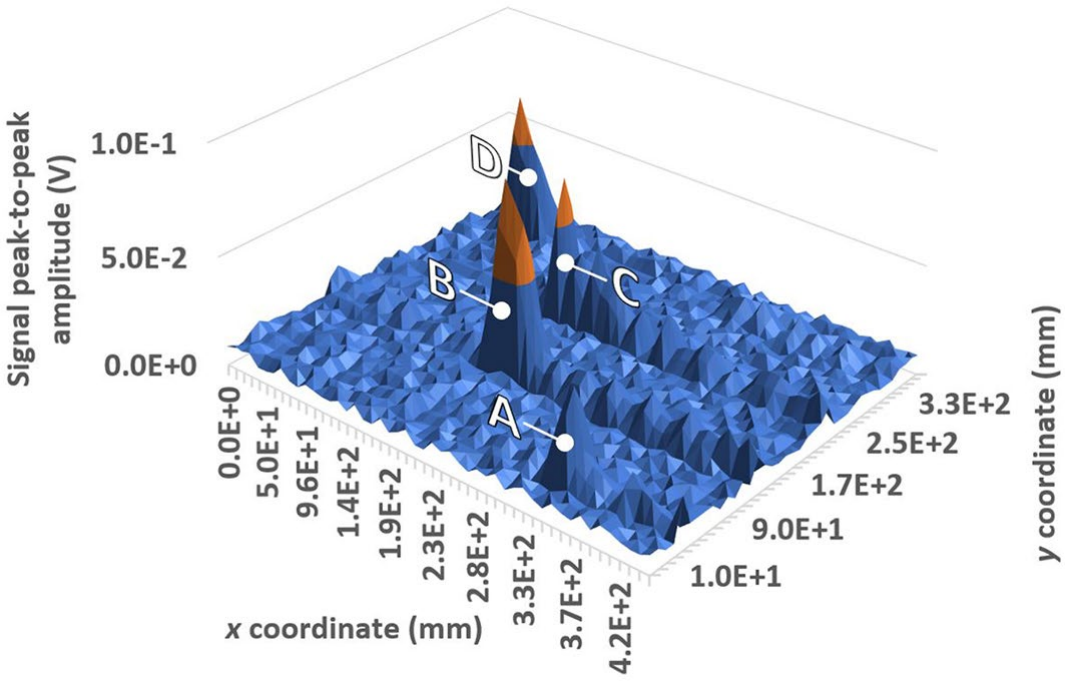
3D map of the plate B-scan, with four peaks associated with the defects, all correctly detected and highlighted following the nomenclature in Fig. 8.

It is also observed that, after the defect, several long-lasting peaks are visible. These peaks are due to the interaction of the SAW wave with the defect. The defect has a depth of 3 mm, less than the wavelength, which is approximately 5.7 mm in the air. The defect has an extension of about 20 mm and the SAW wavefront emitted by the piezoelectric probe reaches the defect with a width greater than the defect, because of the wavefront divergence. This causes part of the wave to pass through the defect, both below and to the side, with diffraction phenomena producing the signals which are visible after the first echo. Figure 11 shows the B scan of the plate (32 ensemble averages for each acquisition). To better visualize the signals deriving from the defects, the signals have been isolated before the back wall echo, so as not to display it on the 3D map. The four defects have been all correctly detected.

**Figure 12:**
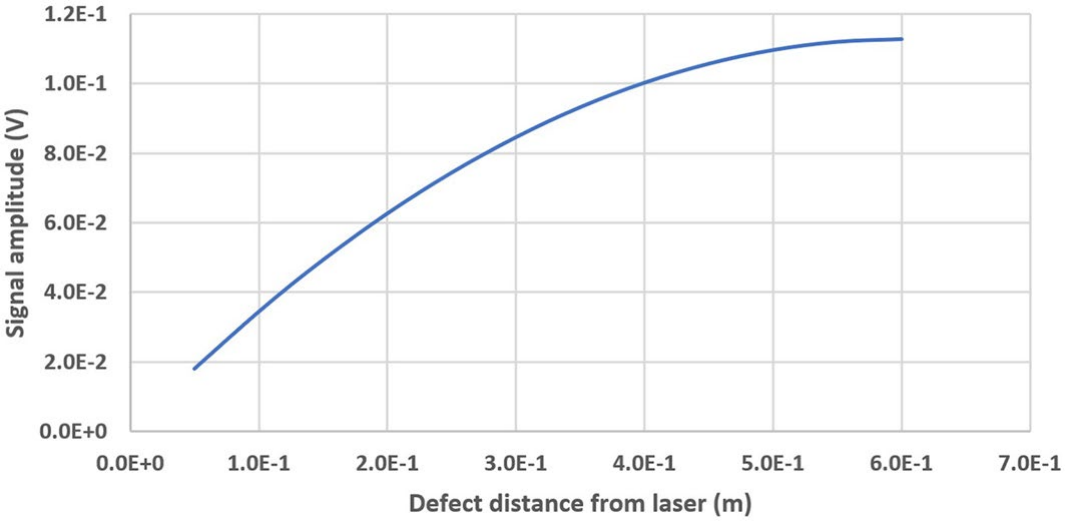
Trend of the photodetector response as a function of the distance between defect and laser source (namely, as $x_s$ increases).

The amplitude of the echo signal is different based on the defect position on the plate. The GCLAD response depends in fact on the beam displacement in correspondence of the photodetector, affected by several factors:

- Distance $x_1$ between the defect and the photodetector and distance $x_s$ between the defect and piezoelectric probe, as highlighted in Eq. (2). Figure 12 highlights the trend of $\varDelta V$ as a function of the $x_1$ and $x_s$ distances (refer to Fig. 1), obtained by Eq. (3) with $\varDelta z$ values given by Eq. (2) and the values of the beam diameter provided by Caron^26^:
  4 $$\begin{aligned} w(x)=w_{0} \sqrt{1+\left( \frac{\lambda x}{\pi w_{0}^{2} n}\right) ^{2}}, \end{aligned}$$
  where $w_0$ is the original waist of the beam, *x* is the distance along the optical path, $\lambda$ is the wavelength of light, and *n* is the index of refraction^45^. Calculation considers *G* = 100, *R* = 100 $\varOmega$, $\kappa$ = 0.119 A/W, *P* = 133 mW, *d* = 6 mm, *t* = 0.25 mm, $w_0$= 0.5 mm, $\lambda$= 6.8 mm, $\delta$= 1 nm. The system sensitivity (i.e., to increase the response of the photodetector) increases as $x_s$ increase. Moreover, with high values of $x_s$, higher deflection angles are obtained and the influence of $x_1$ on the response of the system is greater. Hence, defects which are closer to the laser source, based only on this dependence, would produce a lesser response;
- intensity of the echo. Defects closer to the piezoelectric source intercept a greater percentage of the ultrasonic wavefront; therefore, the echo has higher amplitude and pressure in the air by the refracted wave is greater. In the employed experimental arrangement (see Fig. 6), the defects which are closer to the laser source produce a greater response, based only on this dependence;
- divergence of the laser beam. As evidenced by the authors in a previous research^29^, the sensitivity of the GCLAD depends on the relationship between beam diameter and wavelength in air. The smaller this ratio, the greater the response of the system. In the experimental layout (see Fig. 6), the beam waist is achieved approximately in the middle of the plate;
- distance between the laser beam and the surface of the piece. In the employed experimental arrangement, a defect closer to the piezoelectric source makes the ultrasonic path in steel shorter. As a consequence, also the path in the air for the refracted waves is shorter. Therefore, accounting only for this dependence, defects closer to the laser source would produce a greater response analogously to point (b).

**Figure 13:**
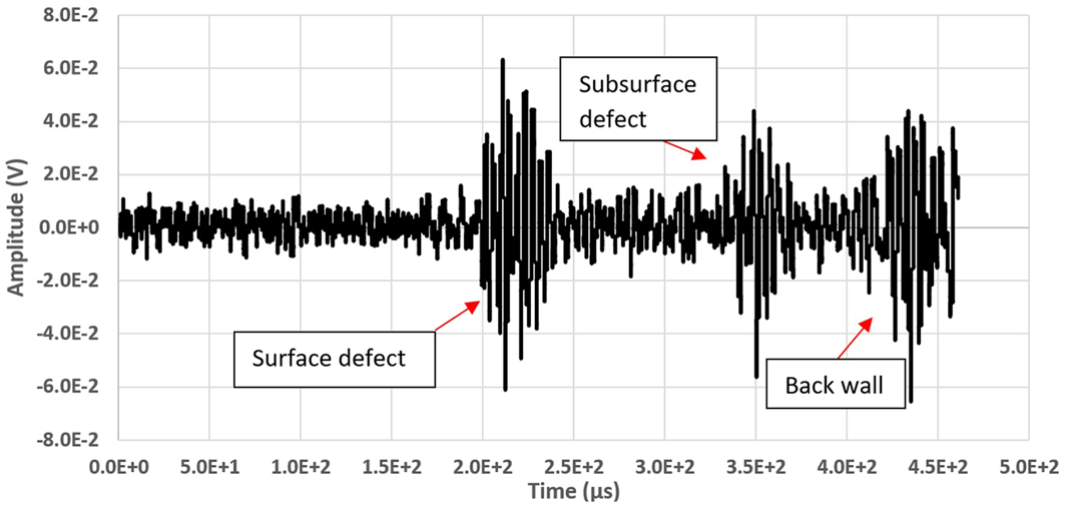
Signal acquired along a line on which both a surface and a subsurface defect are present; the echoes associated with to the two defects and that by the backwall are visible.

To verify the system’s ability to detect even sub-surface defects, further notches have been obtained on the lower face of the plate, as shown in Fig. 8. The defects were practiced on the same scan lines as the upper defects, so that they could be simultaneously identified. Figure 13 depicts a signal (32 ensemble averages) acquired in correspondence of a line where the two defects are present, one on the upper face and the other on the lower face of the plate.

**Figure 14:**
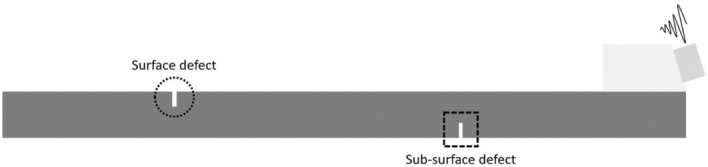
Simulation layout replicating the experimental configuration in Fig. 9.

To understand the generation mechanism and the type of waves present within the ultrasonic signal in Fig. 13, numerical simulations have been carried out. In fact, the interaction between SAWs, the specific surface/subsurface defects, and the fluid domain (where the GCLAD detection occurs) is not documented in the plate inspection-related literature. The simulation layout is shown in Fig. 14 for the 2D solution of the visco-elastic linear wave propagation problem. The Wave 2000^©^ special-purpose software (https://www.cyberlogic.org/wave2000.html) is used to simulate the propagation of longitudinal waves in the plexiglass wedge, whose mode varies at the interface with the metal component generating SAWs. The wave excited by the probe consists of an exponential damped pulse: in five periods, the amplitude of the 500 kHz oscillation is reduced to 10% of its maximum value. A 2D simulation is sufficient to highlight the patterns of the direct and reflected SAWs, as well as their refraction in the air: along the path, a limited amount of energy propagates transversally to the primary wave direction, so that a 3D analysis would unnecessarily complicate the numerical model without adding significant information.

**Figure 15:**
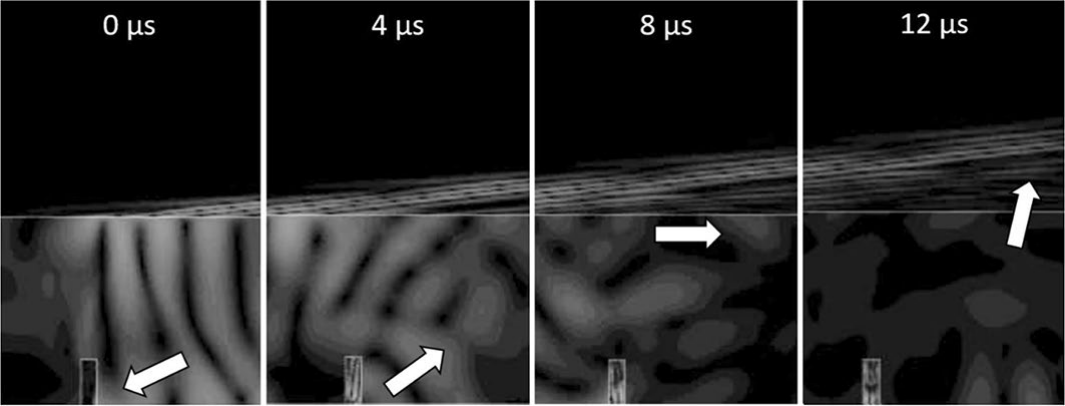
Simulation of SAW propagation in proximity of the subsurface defect position, at different instants. The arrows indicate the position of the ultrasound reflected by the defect at the different time steps.

The SAWs propagating in the metal first encounters the subsurface defect, on which they are reflected as illustrated in Fig. 15: considering the instant 0 $\upmu$s as the instant in which the SAW reaches the defect, part of the ultrasonic energy is reflected resulting in generation of bulk waves which propagate in various directions (4 $\upmu$s). When the bulk wavefront reaches the upper surface of the piece (8 $\upmu$s), a mode conversion occurs that gives rise to SAWs which propagate in the opposite direction compared to the original SAWs, refracted in the air at an angle of − 6.8$^\circ$.

**Figure 16:**
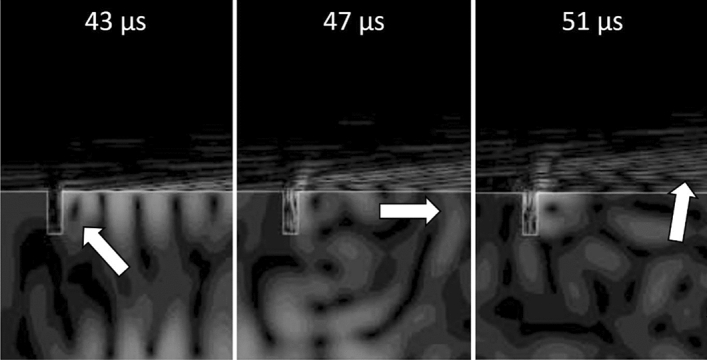
Simulation of SAW propagation in proximity of the surface defect position, at different instants. The arrows indicate the position of the ultrasound reflected by the defect at the different time steps.

The phenomena highlighted in Fig. 15 are retrieved also in correspondence of the surface defect (Fig. 16), in which the SAWs are reflected by the transversal surface of the defect (43 $\upmu$s) and the echo propagates in the opposite direction compared to the original wave (47 $\upmu$s), being subsequently refracted in the air (51 $\upmu$s).

**Figure 17:**
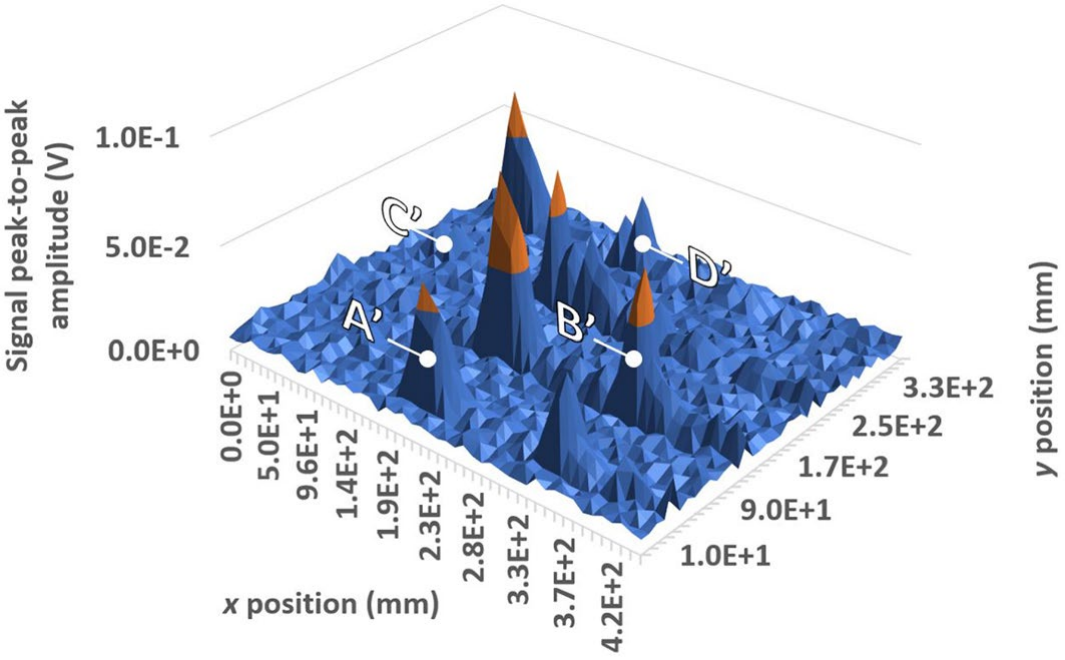
3D map of the plate B-scan, with the eight peaks associated with the surface and subsurface defects all correctly identified (subsurface defects are evidenced following the nomenclature in Fig. 8).

Figure 17 shows the B-scan map obtained for the plate after introducing subsurface defects (32 ensemble averages for each acquisition). All surface and subsurface defects, with a depth lesser than the acoustic wavelength (approximately half), have been correctly identified. Similar considerations to those for the surface scanning of surface defects alone apply regarding the relative amplitude of the detected signal peaks. For what regards the inspection time, let us refer to an analogous monitoring process performed by an ACT. This requires to move the ACT along both *x* and *y* directions rather than solely along *y*, as in the case of the GCLAD. Employing the GCLAD, the time for inspection will be hence reduced of 50%. An increase in the reliability of the measurement will also result, since the movements of the apparatus (and related inaccuracies) are minimized.

## Conclusions

Gas-Coupled Laser Acoustic Detection (GCLAD) is an optical, non-contact ultrasonic detection technique unestablished in the field of non-destructive inspections, yet potentially interesting for its features as a line inspection technology. The GCLAD technique is based on the deviation and displacement sustained by a laser beam when crossing a pressure field in the air, attributable to an ultrasonic wave refracted in the air from the surface of a component under examination. This displacement and deviation of the beam is transduced in voltage by a position-sensitive photodetector. Some previous works have applied the GCLAD technique to ultrasonic detection on mechanical components, but without investigating the actual capabilities of the system regarding defect identification.

Referring to a case study of application to a metal plate, this work highlights that line inspection is possible and effective, as well as detection of surface or subsurface defects oriented perpendicular to the direction of propagation of surface acoustic waves (SAWs). The waves reflected by surface and subsurface defects propagate on the surface and refract in the air, propagating in the opposite direction to that of the direct SAWs; the consequent experimental positioning of the GCLAD laser beam as to be perpendicular to this direction allows simultaneously highlighting the perturbations caused by surface or subsurface defects located along the inspection line.

Unlike point detectors such as piezoelectric probes in air or interferometers/vibrometers, the GCLAD probe laser beam insists on an extended region of fluid above the component, allowing detection of oscillations present along the entire line. By this solution, the plate inspection is based on the relative movement of the piece/instrumentation in a single direction, instead of the two-dimensional movements of the source and receiver required for traditional point monitoring. This instance results in a double benefit, namely a significant simplification of the motion systems for the instrumentation or the component, as well as a contextual reduction in the time required for the structure non-destructive monitoring.
